# Characterization of Macroglia Response during Tissue Repair in a Laser-Induced Model of Retinal Degeneration

**DOI:** 10.3390/ijms24119172

**Published:** 2023-05-24

**Authors:** Laura Jahnke, Souska Zandi, Ahmed Elhelbawi, Federica Maria Conedera, Volker Enzmann

**Affiliations:** 1 Department of Ophthalmology, Inselspital, Bern University Hospital, University of Bern, 3010 Bern, Switzerland; laura.jahnke@extern.insel.ch (L.J.); souska.zandi@insel.ch (S.Z.); 2 Department of BioMedical Research, University of Bern, 3008 Bern, Switzerland; 3 Graduate School for Cellular and Biomedical Sciences, University of Bern, 3012 Bern, Switzerland; ahmed.elhelbawi@unibe.ch; 4 Department of Chemistry, Biochemistry and Pharmaceutical Sciences, University of Bern, 3012 Bern, Switzerland; 5 Department of Medicine, University of Fribourg, 1700 Fribourg, Switzerland; federica.conedera@unifr.ch

**Keywords:** laser injury, Müller cells, astrocytes, retinal degeneration, endogenous regeneration

## Abstract

Reactive gliosis is a hallmark of chronic degenerative diseases of the retina. As gliosis involves macroglia, we investigated their gliotic response to determine the role of S100β and intermediate filaments (IFs) GFAP, vimentin, and nestin during tissue repair in a laser-induced model of retinal degeneration. We validated the results with human retinal donor samples. Experiments were performed in zebrafish and mice using an argon laser (532 nm) to induce focal lesions in the outer retina. At different time points following injury induction, the kinetics of retinal degeneration and regeneration were assessed using hematoxylin and eosin staining (H&E). Immunofluorescence was performed to evaluate Müller cell (GS) and astrocyte (GFAP) injury response and to distinguish between both cell types. Additionally, staining was performed in human retinal sections containing drusen. Focal laser treatment elevated the expression of gliotic markers in the area of the damage, which was associated with increased expression of S100β, GFAP, vimentin, and nestin in mice and humans. In zebrafish, we detected S100β at the first time point, but not GFAP or nestin. Double-positive cells with the selected glia markers were detected in all models. However, in zebrafish, no double-positive GFAP/GS cells were found on days 10 and 17, nor were S100β/GS double-positive cells found on day 12. Macroglia cells showed a different pattern in the expression of IFs in degenerative and regenerative models. In particular, S100β may prove to be a target for suppressing chronic gliosis in retinal degeneration.

## 1. Introduction

Macroglia of the retina—astrocytes and Müller cells—are actively involved in the damage response to several harmful mechanisms (e.g., injury, degeneration, and toxic insults). These pathogenic stimuli can activate macroglial cells and induce reactive gliosis, which can be protective but also become chronic. Initially, reactive changes protect the retina against damage, promoting tissue repair, limiting retina remodeling, and rebalancing neuromodulators, water, and ions [1,2,3]. However, if glia–neuron interactions are altered during a gliotic response, photoreceptor death begins, as can be observed in age-related macular degeneration (AMD), among other diseases [4,5,6]. This process disturbs retinal acid/base, ion, and water homeostasis, and interactions of photopigment and neurotransmitter recycling between glial cells and neurons are lost [2].

Müller cells are the most abundant glia in the vertebrate retina [7]. These cells are involved in the homeostasis, synthesis, and storage of transporters and neurotransmitters, such as glycogen [8,9]. Müller cells are the largest cell type in the retina, spanning the space between the inner and outer limiting membranes (ILM and OLM, respectively). Their cell bodies lie in the inner nuclear layer (INL), their end feet lie at the inner surface of the retina, and they terminate at the vitreous body. Therefore, these cells provide structural stability to the retina [3]. Their projections envelop the cell bodies of the photoreceptors, and they are, therefore, the first to react upon insults, which seems necessary for rapid communication and activation of Müller cells into the protective stage [10,11]. However, as Müller cells are in contact with every cell type in the retina and are necessary for both neuronal and vascular function and viability, their ablation leads to photoreceptor degeneration, vascular leakage, and intraretinal neovascularization [12]. In zebrafish, Müller cells have been found to re-enter the cell cycle upon injury or disease and regain stem cell-like characteristics. In this process, newly formed neurons are generated and integrated into the retinal structure. This stem cell characteristic has not yet been observed in mammals [3,13,14,15,16]. Additionally, in responses to disease and injury, co-cultured Müller cells showed a highly bi-directional response to activated microglia immune cells associated with adaptive and neuroprotective effects [17]. Through expression of adhesion molecules in activated Müller cells, microglia are assumed to activate processes necessary for wound healing [17].

Astrocytes are lower in number than Müller cells, and they can enter the retina through the optic nerve from the brain. They are mainly located in the nerve fiber layer (NFL) and ganglion cell layer (GCL) in most mammals. Astrocytes exhibit a flattened cell body and a series of fibrous, radiating processes encircling endothelial cells [18]. Despite the increasing understanding of astrocytes in the retina, researchers have yet to understand their full functions. Thus far, their crucial role in retinal vascularization and in ion homeostasis (to prevent overexcitation of neurons) has been confirmed [11,12,19]. Additionally, they perform supplementary functions required for the development of the retinal vascular network [19].

As different as they are phenotypically, Müller cells and astrocytes are similar in some functions in the retina. Under physiological conditions, both cell types provide an impenetrable blood–retinal barrier by tightly surrounding the vessels with their projections [20]. They also serve as a link for metabolic exchange between compartments, such as blood vessels, the subretinal space, and the vitreous chamber. Additionally, they influence the synaptic activity of neurons through neurotransmitter recycling [20,21].

**Figure 1:**
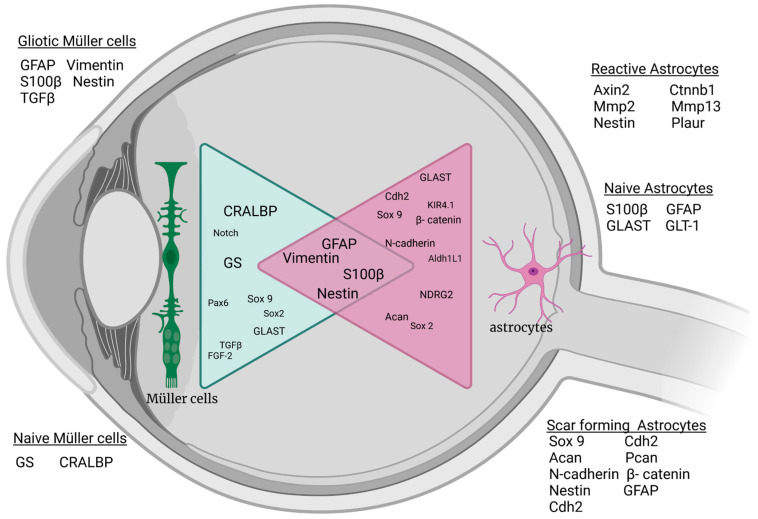
Schematic of common macroglial markers under naive and stressed conditions in the vertebrate retina. Changes in Müller cells and astrocytes after injury or stimulation show similar marker expression for gliotic response. Acan: aggrecan; Aldh1L1: Aldehyde Dehydrogenase 1 Family Member L1; Axin 2: axis inhibition protein; Cdh2: cadherin 2; CRALBP: Retinaldehyde-binding protein; Ctnnb1: Catenin beta 1; FgF-2: basic fibroblast growth factor; GLAST: glutamate A spartate transporter; GLT-1 glutamate transporter-1; GS: Glutamine synthetase; Kir4.1: ATP-dependent inwardly rectifying potassium channel Kir4.1; Mmp13: matrix metalloproteinase 13; Mmp2: matrix metalloproteinase 2; NDRG2: N-Myc Downstream-Regulated Gene 2 Protein; Pax 6: Paired box protein 6; Plaur: plasminogen activator urokinase receptor; S100β: S100 calcium-binding protein B; Sox2: sex-determining region Y-box 2; Sox9: sex-determining region Y-box 9; TGF-β: transforming growth factor β; and GFAP: glial fibrillary acidic protein. Created with BioRender.com.

Both retinal astrocytes and Müller cells show gliotic characteristics during pathophysiological changes and alter the expression of intermediate filaments (IFs), which are sensitive indicators for the gliotic response [1,2]. IFs are cytoskeletal components of cells responsible for structural and mechanical support, and the expression of different IF proteins is tissue- and cell type-specific and dependent on age and activation status [20,21,22,23,24,25,26]. Müller cells and astrocytes express similar IFs, such as glial fibrillary acidic protein (GFAP), vimentin, and nestin. However, their expression patterns differ in time and phase (Figure 1) [24,25]. Pathological changes in the retina trigger the activation and migration of Müller cells to the site of damage. These proliferating cells upregulate the synthesis of GFAP and vimentin [3,27,28,29]. As Müller cells upregulate GFAP in response to injury and in neurodegenerative diseases, GFAP serves as a sensitive marker for retinal stress and damage. In contrast, astrocytes express GFAP under not only reactive but also physiological conditions [4,30,31]. Although macroglia cells are known to be involved during retinal degeneration and in the formation of the glial scar, it is currently unknown to what extent Müller cells and astrocytes participate in gliotic scarring. The main limitation to identifying the involved cell type in detail lies in the fact that both macroglia types express the same IFs, which makes differentiation of their involvement during gliosis challenging [4,8,32,33,34].

To the best of our knowledge, longitudinal studies have focused on the observation of retinal gliosis; none have directly compared degenerative/regenerative changes in Müller cells and astrocytes in the retina. Only one study with a rat model showed in a transgenic model changes in the number and morphology of glial cells with a focus on GFAP expression in astrocytes [18]. In this work, we investigate which macroglia cells play a role in the gliotic response by comparing mouse, zebrafish, and human retinas. We used focal laser photocoagulation to induce retinal damage in the outer nuclear layer (ONL) without Burch’s membrane rupture. While laser injury can induce similar photoreceptor damage to AMD, the clinical pathogenesis of AMD differs from that of laser injury. As glial cells are less sensitive to light than to chemical issues, our laser model was suitable for studying the gliotic response and the IF protein expression of macroglia during wound healing. To distinguish the two types of macroglia, co-staining of Müller cell-specific markers glutamine synthetase (GS) and retinaldehyde-binding protein (CRALBP) with IFs was performed. These proteins are exclusively found in Müller cells and remain unchanged after laser injury [35], whereas adult astrocytes express no GS nor CRALBP in the retina [36]. Thus, we were able to show the extent to which these cell types were involved during degeneration and regeneration.

## 2. Results

### 2.1. Pathological Changes in Retinal Tissue Repair in Zebrafish and Mouse after Laser Injury

**Figure 2:**
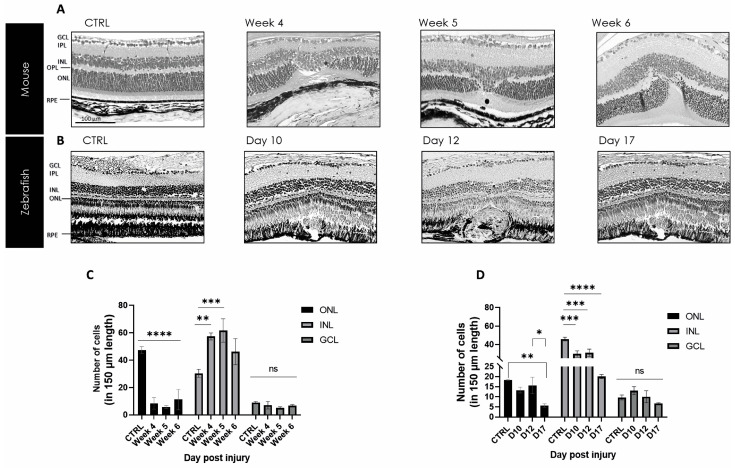
Mouse and zebrafish kinetics of tissue repair. (**A**) Scar formation is seen in the damaged area between week 4 and week 6 post-injury in mouse. (**B**) From day 10 to day 17, post-injury, a wound is formed with complete regeneration in zebrafish. Scale bar: 50 μm. (**C**,**D**) Thickness measurement in different retinal layers for mouse and zebrafish after laser damage in comparison to non-lasered retina. The analyzed length of the retina represents 150 µm corresponding to the lasered damaged area. Significant differences in structural changes between controls and the different time points are depicted as mean ± SEM (*n* = 3 for each time point for each combination) and * *p* < 0.05, ** *p* < 0.01, *** *p* < 0.005 and **** *p* < 0.001 determined with a two-tailed Student’s *t*-test; ns = not significant. GCL, ganglion cell layer; IPL, inner plexiform layer; INL, inner nuclear layer; OPL, outer plexiform layer; ONL, outer nuclear layer; ROS, retinal outer segments; and RPE, retinal pigment epithelium.

H&E overview staining visualized the tissue restoration in zebrafish and mice over time. In a healthy state (CTRL), the structural layer similarity of the visual systems of both animals is shown and can be compared with each other (Figure 2). Additionally, the late stage of tissue repair at week 4, week 5, and week 6 was stained for mice, as was day 10, day 12, and day 17 for zebrafish, respectively. The retinal–choroidal complexes in the mouse samples depict the laser-induced lesion without rupture of Bruch’s membrane. However, in damaged mouse retinas, the considerable loss of photoreceptors within the ONL could be linked to the appearance of unspecific scar tissue to fill up the empty space of detached and degraded neurons (Figure 2A). The images of the zebrafish over the selected time points demonstrate retinal regeneration with the complete reconstruction of the damaged area (Figure 2B). Between mice and zebrafish, the healing process appears different. In mice, we found permanent scar formation in the lesion area, whereas in zebrafish, we see an extensive amount of regeneration. However, the thickness measurements revealed differences in the ONL and INL in both animal models but no significant changes in the GCL (Figure 2C,D).

### 2.2. Regenerating Retina of Zebrafish Does Not Show Gliotic Response in Late-Stage Tissue Restoration

**Figure 3:**
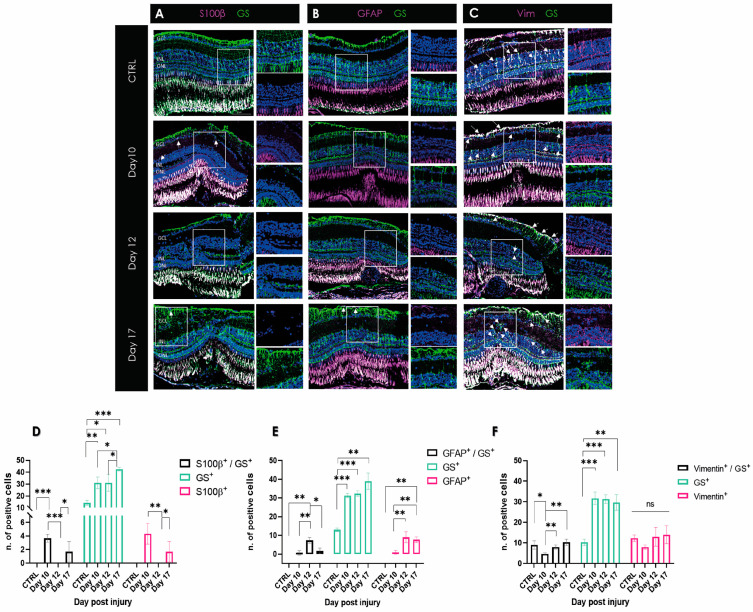
Visualization of macroglia response during wound healing in zebrafish retina after laser damage. (**A**) S100β (magenta) and GS (green) expression over time in zebrafish retinal samples from day 10, day 12, and day 17 post-injury. (**B**) GFAP (magenta) and GS (green) expression over time in zebrafish retinal samples during wound healing. (**C**) Vimentin (magenta) and GS (green) expression over time in zebrafish retina sections. (**D**–**F**) Histograms depicting the respective distribution of single- or double-positive cells (white signal; marked with an arrow). No S100β or GFAP-positive cells were found in the control samples. CTRL, control; GCL, ganglion cell layer; INL, inner nuclear layer; ONL, outer nuclear layer. Data are presented as mean ± SD (*n* = 3 for each time point for each combination) and significance of * *p* < 0.05, ** *p* < 0.01, and *** *p* < 0.001 with a two-tailed Student’s *t*-test. ns = not significant. Scale bars: 50 µm.

To determine the distribution of Müller cells and astrocytes in the lasered zebrafish retina, double immunofluorescence staining was performed on day 10, day 12, and day 17 following damage, using antibodies against GS combined with S100β, vimentin, and GFAP antibodies, respectively. Few to no single- or double-positive S100β cells were located around the damaged area on day 12 pi (Figure 3A). The quantification showed that S100β expression instantly decreased during the retinal repair, beginning from the earlier time point toward the later one (Figure 3D). In contrast, vimentin-positive cells were detected in the inner layer of the retina, and the number of single-positive cells remained not significantly changed (Figure 3C,F). Numerous double-positive vimentin and GS cells were observed in the retina on all selected days as well as in the control tissue, with increasing numbers over time (*p* < 0.01) (Figure 3C,F). However, only low GFAP expression could be shown in the late stage of regeneration in zebrafish (Figure 3B,E). Especially in the middle of tissue restoration on day 12 pi, we could see a peak of double as well as single-positive GFAP cells (Figure 3E).

### 2.3. Laser Injury Increased Expression of S100β and GFAP in the Gliotic Mouse Retina

**Figure 4:**
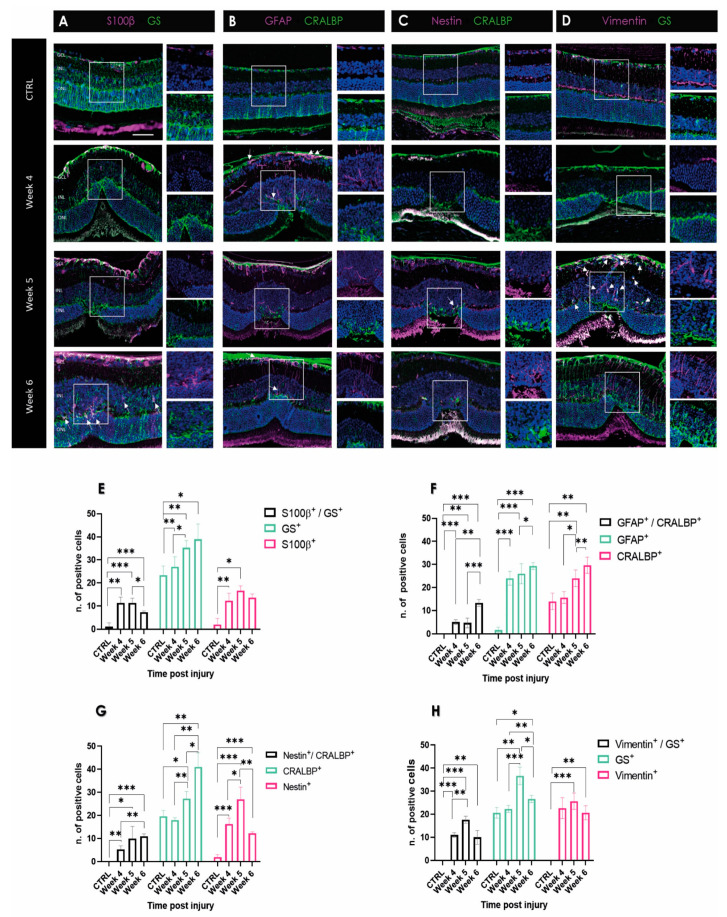
Changes in expression of makers of reactive gliosis in mouse retina during degenerative processes following laser injury. Images of 5 μm paraffin sections are shown in each panel from various time points after laser. Scale bar: 50 μm. The control section represents a retina without any laser injury. (**A**) S100β (magenta) and GS (green) expression over time in mouse retinal samples during wound healing. (**B**) GFAP (magenta) and CRALBP (green) expression over time in mouse retinal samples during wound healing. (**C**) Nestin (magenta) and CRALBP (green) expression. (**D**) Vimentin (magenta) and GS (green) expression over time from week 4, week 5, and week 6 post-injury in mouse retina. Disorganization can be seen in the ONL in week 5 and week 6 pi. The ONL is not rearranged and shows scar tissue. Double-positive cells show white signal and are marked with an arrow. (**E**–**H**) Quantification of detected single- or double-positive cells (white fluorescence signal). No vimentin-positive cells were found in the controls. CTRL, control; GCL, ganglion cell layer; INL, inner nuclear layer; and ONL, outer nuclear layer. Data are presented as mean ± SD (*n* = 3 for each time point for each combination) and significance of * *p* < 0.05, ** *p* < 0.01, and *** *p* < 0.001 with a two-tailed Student’s *t*-test.

In order to investigate a potential time-dependent change in Müller cells and astrocyte involvement in scar formation in the mouse retina, we compared the morphologies of the different macroglial cell types stained with different markers in the late stage of scar formation (Figure 4A–D). Double staining showed GS- and S100β-positivity in Müller cell endfeet in the GCL at week 5 and week 6 after laser-induced retinal damage. Although both proteins can be detected at the same location, there are single-positive cells with no overlap indicating two distinguishable cell types. In healthy retinas, GFAP staining was absent throughout the retina (Figure 4B). At weeks 5 and 6 post-injury, GFAP-positive cell processes were arranged in clusters, forming characteristic shooting star-like structures (Figure 4B). Additionally, individual single GFAP-positive cells with a stellate-shaped form were visible, which were laid like a plaster over the scar (Figure 4B). These cells were also visualized in the IPL and INL. This particular distribution of processes in the ONL was not present at week 6 post-injury.

The vimentin-positive cells showed comparable distribution in a firework-like array of cells, mainly in the scar at week 6 (Figure 4D). This differs from the control, where a positive signal is seen throughout the whole OPL of the retina. In addition, we found a difference in gliotic markers in the area of the scar within a week. At week 5, single GS-positive cells were found, as well as the previously mentioned firework-like positive vimentin Müller cells. Single vimentin-positive cells were visualized from the GCL through the IPL to the ONL (Figure 4D), compartmentalizing the scar from healthy tissue.

### 2.4. Activation of Macroglia in Human Retina with Early Degeneration (Drusen)

**Figure 5:**
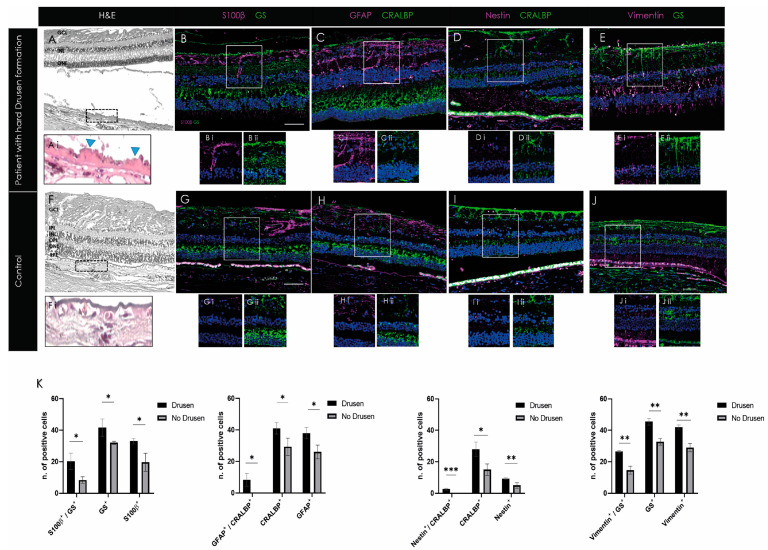
Gliotic response in human retinal samples showed activation of macroglia in retina containing drusen. (**A**–**J**) Individual cells showed elongated fibrillar projections in an irregular arrangement with S100β and GFAP expression. In addition, significantly higher CRALBP and GS-positive cells were observed in drusen samples. (**K**) Histograms depict the mean ± SD of the number of single or double-positive cells (white signal; marked with an arrow) expressing gliotic and Müller cell-specific markers in drusen-positive compared to control retinas. GCL, ganglion cell layer; IPL, inner plexiform layer; INL, inner nuclear layer; OPL, outer plexiform layer; ONL, outer nuclear layer; and RPE, retinal pigment epithelium. Data are presented as mean ± SD (*n* = 3 for each time point for each combination) and significance of * *p* < 0.05, ** *p* < 0.01, and *** *p* < 0.001 with a two-tailed Student’s *t*-test. Scale bars: 50 µm.

In order to investigate if the data from the mouse model correspond to the characteristics of retinal degeneration in patients, three human retina samples containing drusen and three samples without drusen were stained with the selected gliotic markers (vimentin, nestin, GFAP, and S100β). To visualize the degree of drusen formation, each sample was also H&E stained. The area of the drusen between the RPE basal lamina and the inner collagen layer of the RPE layer was enlarged for highlighting (Figure 5A,E). It can be seen that the selected samples contain hard drusen (Figure 5A). In contrast, no drusen were seen in the controls (Figure 5E). Single-positive S100β cells were observed in the IPL and the OPL. This was not seen in non-drusen samples (Figure 5B). Similarly, increased GFAP single-positive cells were seen in the retina of drusen-containing samples (Figure 5C). Individual cells showed elongated fibrillar projections in an irregular arrangement (Figure 5B). GFAP-positive cells were also found in the controls, but their number was significantly lower (Figure 5C,H; *p* < 0.05). A significant difference in CRALBP and GS-positive cells was observed between the drusen and non-drusen samples. Interestingly, nestin was detected more abundantly in drusen-positive retinas between Bruch’s membrane and RPE layer in drusen samples (Figure 5C). Controls showed no nestin or GFAP double-positive cells. Quantification emphasized the significant increase in double as well as single-positive cells in the drusen-positive donor samples compared to the non-drusen donors of all selected gliotic markers (Figure 5K).

## 3. Discussion

The gliotic response differs between vertebrates with high or minimal/absent regenerative capacity; thus, cross-species comparison of gliosis is of great interest. A common feature of retinal gliotic response is characterized by the clustering of glial cells in the damaged area. The cell type mostly known to be involved during gliosis is Müller cells, independent of the vertebrate species and their repair ability [3]. During reactive gliosis, Müller cells express GFAP, nestin, vimentin, or/and S100β expressions, and thus, those markers are considered indicators for stress reaction after damage. However, astrocytes can also take part in gliosis, and indeed, they have the capacity to upregulate the same IFs. Here, we compared the gliotic response between teleost (zebrafish) with life-long regenerative capacity and mammals (mouse and human) with minimal/absent regenerative capacity. Our data show differing expression of intermediate filaments in the teleost retina compared to the mammalian one and suggest that astrocytes, as well as Müller cells, play a pivotal role in retinal gliosis.

Herein, we compared retinal tissue repair between an animal model with high regenerative capacity (zebrafish) and one with low regenerative capacity (mouse) to demonstrate differences in the gliotic response among vertebrates. For this purpose, we used the laser injury model to produce rapid and focal damage to the retina located in a well-defined area of the ONL. The model can reproducibly induce processes that characterize retinal degeneration. In order to induce injury in the zebrafish retina comparably to the mouse, we modified the laser setting by reducing the laser pulse to the lowest value. With that, the pulse was three times lower compared to the laser model of Dicicco et al., 2014 [37]. Still, even with the lowest pulse setting, the rupture of Bruch’s membrane cannot be excluded completely. The newly implemented settings led, however, to the prolongation of the regenerative pathway, unlike previously published [38]. Additionally, the laser model has many advantages compared to chemical, mechanical, and thermal injury methods, as the damage is readily identified with OCT, collateral damage in the retina is minimal, and changes can be observed in a defined area.

It is known that glial cells have a critical role in the response of the central nervous system to injury. Reactive gliosis is thought to represent a cellular attempt to protect the tissue from further damage, but in case of overreaction, it limits tissue remodeling, and the process becomes chronic. In response to the retinal injury, both macroglia cell types become rigid by reactive gliosis with upregulating GFAP and other intermediate filaments together with morphological and functional changes [11,19]. The exact function of GFAP remains an enigma, and all mutant mouse models (GFAP knockout or overexpression) of the past showed normal development and fertility [39]. Generally, all IF type III proteins provide mechanical support for the plasma membrane to contact other cells or the extracellular matrix, and are also involved in the function of the cell’s cytoskeleton [40]. The most widely shared assumption is that this occurs primarily in Müller cells. Additionally, the upregulation of vimentin, GFAP, nestin, and S100β are hallmarks of gliotic response in both retinal macroglia [18,41].

Immunohistochemical data revealed a high variability among individual macroglia cells with respect to their IF expression. A ‘‘conservative’’ or non-proliferative gliosis is characterized by increased expression of IFs, such as GS and CRALBP, in moderate transient or non-proliferating Müller cells with normal functions [42]. However, it is known that different retinal diseases lead to an increase or decrease in GS expression in Müller cells. For example, glutamate transporters such as GS are downregulated in glaucoma, ischemia, and diabetic retinopathy [2]. The role of CRALBP during gliotic response is still not completely understood. As CRALBP is involved in visual pigment cycling, CRALBP-positive cells, in general, conceivably act as a retinoid buffer during development in order to protect the retina from retinoid toxicity [36]. GFAP is a highly regulated protein whose expression is induced by multiple factors in different diseases and is the main IF in gliotic response. GFAP-only filaments were observed in CNS. A natural partner for GFAP is vimentin [43], which needs GFAP or nestin in order to polymerize into IFs in non-reactive macroglia [39]. In the absence of GFAP, gliosis can still be induced due to the presence of vimentin only. Firstly, we found GS as well as CRALBP expression in the damaged area in all samples. Secondly, single-positive GFAP cells, not clearly identifiable as Müller cells or astrocytes, were detected. This observation is equally evident in the human samples. Therefore, we hypothesize that Müller cells may have downregulated their GS or CRALBP expression, and their activation after laser injury is indicated by upregulation of GFAP and nestin [44].

Furthermore, in the mouse model, we could identify Müller cells around the damaged area (Figure 4), whereas, in zebrafish, the formation of a characteristic GS-positive scar was not found (Figure 3). In contrast, a decreased number of GS-positive Müller cells was observed in the laser spot compared to the control, suggesting that Müller cells undergo dedifferentiation due to their progenitor character in zebrafish [40]. There are no antibodies commercially available for the detection of CRALBP and nestin (see below) in zebrafish, and thus we could not compare these signals in retinal sections from zebrafish with our data from mice and humans. 

Activation and migration of astrocytes into the scar region should also be considered. In healthy retinas, astrocytes are tightly packed around blood vessels and are exclusively located in the innermost retinal layers [18,45]. The classifications of astrocytes in activated stages are described in the retina as type 1 (inflammatory) or type 2 (ischemic) [46]. Thereby, subtype 1b astrocytes in their quiescent phase are the major cell type in the inner limiting membrane (ILM) and close to the optic nerve [11,47]. This might explain the formation of a permanent scar in the mammalian retina, as type 1b astrocytes are present in the retina and express ECM proteins like elastin during development but also in direct response after mechanical stress [47]. In the quiescent state, all astrocytes are star-shaped. When there is increased migration and proliferation to the damaged retina due to stress, their projections retract and go from the quiescent to the active state [48]. During migration and aggregation, glial cells cannot be clearly differentiated by shape [49]. In this regard, astrocytes and Müller cells with hypertrophic, enlarged soma or thickened processes were found in the mouse model at week 5.

Retinal laser injury stimulated Müller cell activation as indicated by upregulation of the intermediate filament proteins GFAP, vimentin, and nestin. Vimentin is the only IF type found in a wide variety of cells, including astrocytes, Müller cells, fibroblasts, endothelial cells, macrophages, neutrophils, and lymphocytes [25,50]. However, vimentin knockout mice did not reveal a deviant phenotype; the animals grew and reproduced normally. GFAP filaments were also found to be absent in certain glial cells, but transfection of cultured vimentin-lacking astrocytes with a vimentin cDNA restored the GFAP-vimentin filament network, indicating that co-assembly of vimentin and GFAP is pivotal [51]. Our observations in the mouse retina showed increased GFAP as well as vimentin filament formation (Figure 4). This is congruent with the observations of Galou et al., 1996 [49]. However, as one cannot clearly distinguish between the two macroglial cell types, we have not performed double staining of GFAP and vimentin. However, the observed data showed that vimentin-positive cells also express GS and thus were most likely Müller cells. Of great interest was the change between weeks 5 and 6, clearly seen by the formation of the firework-like focus through the ONL in the area of the glial scar. Earlier retroviral experiments with human vimentin antisense cDNA showed that vimentin might play a key role in glial scar formation [50]. In this context, it is interesting to note that in zebrafish, although vimentin is primarily expressed in Müller cells, little GFAP is observed (Figure 3). Thus, in zebrafish, GFAP might influence vimentin expression rather than vimentin modulating GFAP expression, like in mice. Nestin has been observed to be upregulated in the progression of chronic gliosis [25,52,53,54]. Although nestin is now known as a marker for neural stem and progenitor cells, it is less suitable for differentiating glial cells [55]. Both reactive astrocytes and Müller cells, as well as microglial cells, can express nestin under certain conditions. Müller cells express nestin after acute injury, such as laser photocoagulation or pharmaceutically induced retinal degeneration. Similarly, astrocytes express nestin during experimental retinal detachment in rats [56,57]. Our data suggest that nestin is distinctively upregulated in the RPE layer, but not in the sensory retina. In the mouse, however, we see nestin-positive cells, especially at week 5 in the GCL, depicting a distinct difference between the two vertebrate species. However, the number of nestin-expressing cells, most probably astrocytes, appeared to be greater.

As elsewhere in the CNS, immediate changes in retinal glial cells may have both beneficial and detrimental effects on structural and functional regeneration. By direct comparison, the expression of S100β and GFAP was shown to be differently regulated. Both gliosis markers have not been observed in the late phase of the regenerative process in zebrafish (Figure 3). In contrast, both mouse and human samples showed increased expression of S100β (Figure 4 and Figure 5). Single cells spanning the entire retina, clearly identifiable as Müller cells by GS positivity, showed S100β expression near the scar. Astrocytes in the GCL also showed elevated S100β expression. This suggests that this calcium-binding protein is indicative of chronic gliotic scar, which adversely affects tissue restoration. In the past, it has been suggested that high levels of S100β, especially in biological fluids of the brain, were recognized as a reliable biomarker of active neural distress [58,59]. Increasing evidence suggests that at high concentrations, S100β triggers tissue response toward damage in neurons [60]. In Parkinson’s, Alzheimer’s, and other psychiatric disorders, it is an important tool for monitoring disease progression [59]. In particular, damaged astrocytes express S100β and play a role in chronic brain disorders [61]. So far, it is not known how S100β influences Müller cell activity during retinal gliotic response and whether this protein affects the chronic character of the glial scar depending on its concentration during degenerative changes. This protein seems to be an important indicator of degenerative mechanisms in the retina, although less suitable as a marker to distinguish between Müller cell and astrocyte response. However, as the protein was found in the late phase of degeneration, possible therapeutic intervention might be too late.

**Figure 6:**
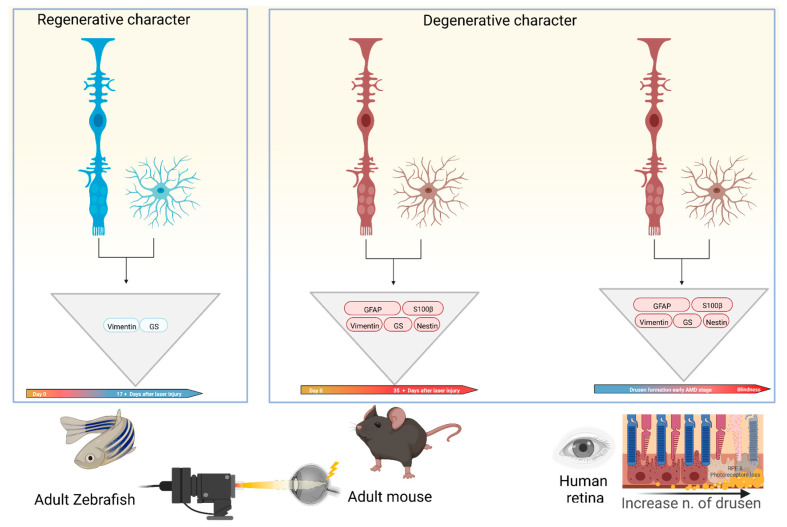
Schematic depiction of gliotic response involving macroglia in the late phase of diseased retinas in different species. Different colors of Müller cells and astrocytes underline the regenerative (blue) or degenerative (red) character of wound healing. In all models, macroglia have the ability to undergo the same gliotic response, but zebrafish do not show expression of all selected intermediate filaments and gliotic markers. This might be the reason for aberrant retinal tissue repair after retinal injury between both animal models. Created with BioRender.com.

In summary, we found differences in the gliotic response after inducing damage to the ONL between species with high and low regenerative capacity. There is a temporal window in which S100β expression is coincident with the upregulation of GFAP and vimentin at week 6 post-injury, pointing to its involvement in the formation of a chronic scar in mammals. In zebrafish, it can be clearly stated that no gliotic scar is detectable late in the course of tissue restoration. Likewise, we show in the mouse model that the data are consistent with the results from human donor samples. This is summarized in Figure 6. Whether and how astrocytes play a role in the formation of retinal scar tissue has not been conclusively determined. In general, glial interactions attempt to maintain retinal homeostasis and regulate each other’s activity. However, glial interactions can also create imbalances and thus contribute to retinal neurodegeneration. To date, there is only sparse literature that deals in detail with the glia–glia interaction, especially with the partnership between Müller cells and astrocytes [62]. However, these interactions are an important topic not only for understanding detailed homeostasis in the healthy retina but also for understanding changes in neurodegenerative diseases such as AMD.

## 4. Materials and Methods

### 4.1. Zebrafish Studies

Adult zebrafish of both sexes (AB strain; >8 months of age) were kept at the animal facility of the Dept. of Anatomy, University of Bern, under standard conditions at 28 °C, 14 h/10 h light/dark cycle. Zebrafish were fed with *Artemia salina* once per day and with dry food twice per day. All experimental procedures were approved by the governmental authorities of the Canton of Bern (BE 34/2019).

To induce laser injury, zebrafish were anesthetized with 0.16% tricaine (Pharmaq, London, UK) and positioned on a custom-made rubber fixation stand. Three laser spots were induced by 532 nm laser photocoagulation (Visulas 532s laser workstation, Carl Zeiss Meditec AG, Oberkochen, Germany). Each injury was induced with the power of 50 mW for 100 ms and the size of 50 μm diameter.

### 4.2. Mouse Studies

Adult C57BL/6J mice (Charles River Germany, Sulzfeld, Germany), male and female, aged 6–9 weeks, were used in the experiments. Animals were kept under standard conditions with 12 h light/dark cycle in individually ventilated cages (IVC) in a temperature-controlled animal facility (Dept. of BioMedical Research, University of Bern). They were fed with standard laboratory chow and water ad libitum. All experimental procedures were approved by the governmental authorities of the Canton of Bern (BE 146/2020).

Mice were anesthetized with 1 mg/kg medetomidine (Dormitor, 1 mg/mL; Provet AG, Lyssach, Switzerland) and 80 mg/kg ketamine (Ketalar, 50 mg/mL; Parke-Davis, Zurich, Switzerland). Pupils were dilated with 2.5% phenylephrine and 0.5% tropicamide (ISPI, Bern, Switzerland). Hydroxypropyl methylcellulose, 20 mg/mL (Methocel 2%; OmniVision AG, Neuhausen, Switzerland), was applied to the eyes to keep them hydrated. To induce retinal damage in the outer nuclear layer (ONL) without rupturing Burch’s membrane, eyes were lasered using 300 μm spot size, 60 ms duration, and 60 mV pulses of a 532 nm laser photocoagulation (Visulas 532s laser workstation with slit lamp, Carl Zeiss Meditec AG). Eight laser spots were centered with 2 to 3 disk diameters from the optic nerve by using a coverslip to allow viewing of the posterior pole of the eye. In order to avoid the induction of choroidal neovascularization (CNV), lesions in which bubbles, a criterion for Bruch’s membrane rupture, were identified during the laser process, were excluded from the study.

### 4.3. Human Samples

The study was carried out on human retinal tissue in a series of three different sections per group with or without early drusen formation (age 70–90). The research complies with the Swiss human research act (HRA), stating that small quantities of bodily substances removed in the course of transplantation may be anonymized for research purposes without consent (HRA chapter 5, paragraph 38). In the case of drusen-positive donors, the retina exhibits micro-drusen (<25 μm) as a single deposit of extracellular material or as a chain-occurring row, or hyalinized round deposits (>25 μm). The selection of the drusen stage is described in detail elsewhere [63]. Donors with any systemic or ocular comorbidities were excluded based on the criteria for corneal donations (i.e., sepsis, meningitis, HIV, lues, hematological neoplasms, all ocular tumors, Creutzfeldt–Jakob disease, rapid progressive dementia or degenerative neurological condition, eye surgery within 6 months or after transplantations, and drug abuses).

### 4.4. Histology and Cell Quantification

Eyes were enucleated on days 10, 12, and 17 (zebrafish) or weeks 4, 5, and 6 (mice) post laser injury (pi), fixed with 4% paraformaldehyde at 4 °C for 24 h and embedded in paraffin. Then, five µm sections of the posterior segment of sagittal-oriented eyes were stained with H&E (hematoxylin (Sigma, St. Louis, MO, USA) and eosin (Roth, Karlsruhe, Germany)). Additionally, four donor retina sections with and without drusen formation were used. Finally, samples were mounted with Eukitt^®^ (Medite Service AG, Dietikon, Switzerland). Images were generated at 20x magnification with a scanning laser microscope (Zeiss LSM710; Carl Zeiss Microscopy, Jena, Germany) and processed using Fiji-win64 (NIH, Bethesda, MD, USA). For H&E, the thickness in the ganglion cell layer (GCL), the inner nuclear layer (INL), as well as outer nuclear layer (ONL) was counted in the lasered area.

### 4.5. Immunohistochemistry

Paraffin tissue sections were also used for immunohistochemistry. For this, sections were incubated in a blocking solution containing 3% normal goat serum (Agilent Technologies, Santa Clara, CA, USA), 0.5% casein (Sigma), and 0.05% Triton X-100 (Sigma) in Tris-buffered saline (TBS; Sigma) at room temperature for 1 h. Primary antibodies diluted in blocking buffer were added at 4 °C overnight (Table 1). This was followed by washing with TBS and 0.05% Triton X-100 and incubation with the respective secondary antibodies (Alexa Fluor^®^ 488 & 594, 1:500; Abcam, Cambridge, UK) and 4, 6-Diamidino-2-phenylindole (DAPI; Sigma) to counterstain nuclei for 3 h at room temperature. All sections were mounted in Inova Mounting Medium (Ruwag Handels AG, Bettlach, Switzerland). Images were also generated at 20x magnification with a scanning laser microscope (Zeiss LSM710; Carl Zeiss Microscopy) and processed using Fiji-win64 (NIH).

### 4.6. Statistical Analysis

Retinal thickness measurements were performed for all groups in three eyes of three animals for each time point. To statistically evaluate morphometric data, the thickness of the retina layers was evaluated with two-way ANOVA followed by Holm–Sidak’s multiple comparisons test. These data are presented as mean ± standard error of the mean (SEM). In order to determine the number of macroglia cells at the different time points, the number of single and double-positive stained cells in the damaged area was quantified. These data are presented as mean ± standard deviation (SD) from three samples. The level for statistical significance was set at *p* < 0.05 (*), *p* < 0.01 (**), *p* < 0.005 (***), and *p* < 0.001 (***); ns = non-significant. Statistical analysis was performed with GraphPad Prism (version 8.0.1; San Diego, CA, USA).

## Figures and Tables

**Table 1:** Antibody list for immunostaining to compare macroglia response in different tissues.

Sample	Protein	Code	Company	Dilution
Zebrafish	GS	ab210107	Abcam	1:1000
		ab16802	Abcam	1:200
	S100β	SC57-02,	Novus Biologicals Zug, Switzerland	1:200
	Vimentin	NBP1-97672	Novus Biologicals	1:200
	GFAP	OPA1-06100	ThermoFisher Scientific Basel, Switzerland	1:200
Mouse/Human	GS	ab64613	Abcam	1:200
		ab16802	Abcam	1:200
	CRALBP	NBP2-58065	Novus Biologicals	1:200
	S100β	SC57-02	Novus Biologicals	1:200
	Vimentin	NBP1-97672	Novus Biologicals	1:200
	GFAP	NBP1-05197	Novus Biologicals	1:200
		OPA1-06100	ThermoFisher Scientific	1:200
	Nestin	NB100-1604	Novus Biologicals	1:200

## Data Availability

Not applicable.
